# Survival of non-transplant patients with multiple myeloma in routine care differs from that in clinical trials—data from the prospective German Tumour Registry Lymphatic Neoplasms

**DOI:** 10.1007/s00277-018-3449-8

**Published:** 2018-08-01

**Authors:** Wolfgang Knauf, Ali Aldaoud, Ulrich Hutzschenreuter, Martine Klausmann, Stephanie Dille, Natalie Wetzel, Martina Jänicke, Norbert Marschner, and the TLN-Group (Tumour Registry Lymphatic Neoplasms)

**Affiliations:** 1 Joint Outpatient-Centre for Oncology, Frankfurt a. M., Germany; 2 Joint Outpatient-Centre for Haematology and Oncology, Leipzig, Germany; 3 Joint Outpatient-Centre for Haematology and Oncology, Nordhorn, Germany; 4 Joint Outpatient-Centre for Haematology and Oncology, Aschaffenburg, Germany; 5 Medical Department, iOMEDICO, Freiburg, Germany; 6 Clinical Epidemiology and Health Economics, iOMEDICO, Freiburg, Germany; 7 Outpatient-Centre for Interdisciplinary Oncology and Haematology, Wirthstrasse 11c, 79110 Freiburg, Germany

**Keywords:** Multiple myeloma, Registries, Outpatients, Outcome assessment

## Abstract

Despite increasing treatment options, multiple myeloma (MM) remains incurable for most patients. Data on improvement of outcomes are derived from selected patient populations enrolled in clinical trials and might not be conferrable to all patients. Therefore, we assessed the trial eligibility, sequential treatment, and survival of non-transplant patients with MM treated in German routine care. The prospective clinical cohort study TLN (Tumour Registry Lymphatic Neoplasms) recruited 285 non-transplant patients with symptomatic MM at start of first-line treatment in 84 centres from 2009 to 2011. Demographic and clinical data were collected until August 2016. Trial-ineligibility was determined by presence of at least one of the common exclusion criteria: heart/renal failure, liver/renal diseases, polyneuropathy, HIV positivity. All other patients were considered potentially trial-eligible. Thirty percent of the patients in our study were classified as trial-ineligible. Median first-line progression-free survival (PFS) and overall survival (OS) of trial-ineligible patients were inferior to that of potentially trial-eligible patients: PFS 16.2 months (95% CI (confidence interval) 11.1–20.4) vs. 27.3 months (95% CI 23.3–33.0); OS 34.2 months (95% CI 21.6–48.1) vs. 58.6 months (95% CI 48.6–64.4). A high percentage of non-transplant patients with MM in German routine care would be ineligible for participation in clinical trials. Despite similar treatment algorithms, their first-line PFS and OS were shorter than those of potentially trial-eligible patients; the survival data of the latter were similar to results from clinical trials. Physicians should be aware of the fact that results from clinical trials may not mirror “real world” patient outcomes when discussing outcome expectations with patients. Trial registration: Clinicaltrials.gov identifier: NCT00889798.

## Introduction

Multiple myeloma (MM) is a malignant plasma cell disorder and accounts for approximately 10% of all haematologic malignancies [1]. Despite increasing treatment options, MM is considered incurable because most patients eventually relapse or become refractory to the treatment. 6510 new cases of MM have been recorded in Germany in 2014; their prognosis is rather unfavourable with a 5-year survival of 47% for women and 49% for men [2]. Upon diagnosis, eligibility for stem cell transplantation is determined by age, performance status, and comorbidities [3]. As MM is predominantly diagnosed at age 65 or greater, the majority of patients are not eligible for transplantation. Furthermore, due to the growing number of elderly individuals in our population, the number of elderly patients with malignancies continues to increase and treatment recommendations for elderly and transplant-ineligible patients are of growing interest.

The proteasome inhibitor bortezomib and the immunomodulatory drug thalidomide have changed the treatment algorithm for elderly patients. The combination therapies bortezomib, melphalan, prednisone (VMP) and melphalan, prednisone, and thalidomide (MPT) were standard of care for non-transplant patients during the study period [4–6]. We previously analysed the first- and second-line treatment of non-transplant patients with MM in routine care in Germany and revealed that 40% of the patients received VMP and 8% MPT as first-line treatment in 2009 to 2011 [7]. Recently, treatment with the combination of lenalidomide with low-dose dexamethasone has yielded promising efficacy and safety results [8]. Combination therapies with novel proteasome inhibitors (like carfilzomib [9], ixazomib [10]), monoclonal antibodies (like elotuzumab, daratumumab [11]), or the histone de-acetylase inhibitor panobinostat [12] may well pave the way for new standards of care for non-transplant patient [13, 14].

However, the results from clinical trials might only partially be conferred to patients in routine care as the patient populations can differ considerably, mainly due to the stringent inclusion criteria of randomised clinical trials. In metastatic renal cell carcinoma, for instance, 35–57% of the patients in routine care would be deemed ineligible for clinical trials, as assessed by common exclusion criteria [15, 16]. Data from prospective, clinical cohort studies like ours are of high importance, as they complement the results from clinical trials and generate data on the tolerability and effectiveness of new therapies in daily practice [17, 18].

Here, we present data from the prospective cohort study TLN (tumour registry lymphatic neoplasms), which recruited a representative sample of non-transplant patients with MM from routine care in Germany. Aim of this study was to assess the potential eligibility of patients for clinical trials according to the presence of common exclusion criteria and to compare the treatment and the progression-free survival (PFS), overall survival (OS), and disease-specific survival (DSS) of trial-ineligible with potentially trial-eligible patients.

## Patients and methods

### Data source

The TLN is an open, longitudinal, multicentre, observational, prospective cohort study, which started in 2009. The study was reviewed by the responsible ethics committee and registered at ClinicalTrials.gov (NCT00889798). 3795 patients aged ≥ 18 years with aggressive or indolent non-Hodgkin lymphoma, chronic lymphocytic leukaemia, or MM at the start of their first- or second-line treatment were recruited into the TLN. The present analysis focuses on 490 patients with systemic treatment of MM recruited between April 2009 and November 2011 in 84 medical oncology centres across Germany. Written informed consent was obtained from all patients. Treatment of patients started within 4 weeks prior or until 8 weeks after signing informed consent. Patients were treated according to physicians’ choice and visits were scheduled individually; no specifications for treatment were imposed at any time. All patients were followed until death, loss to follow-up, withdrawal of consent, or up to 5 years from enrolment. Follow-up was limited to 5 years due to limited funding. The TLN has previously been described in detail [7].

### Cohort definition

**Fig. 1:**
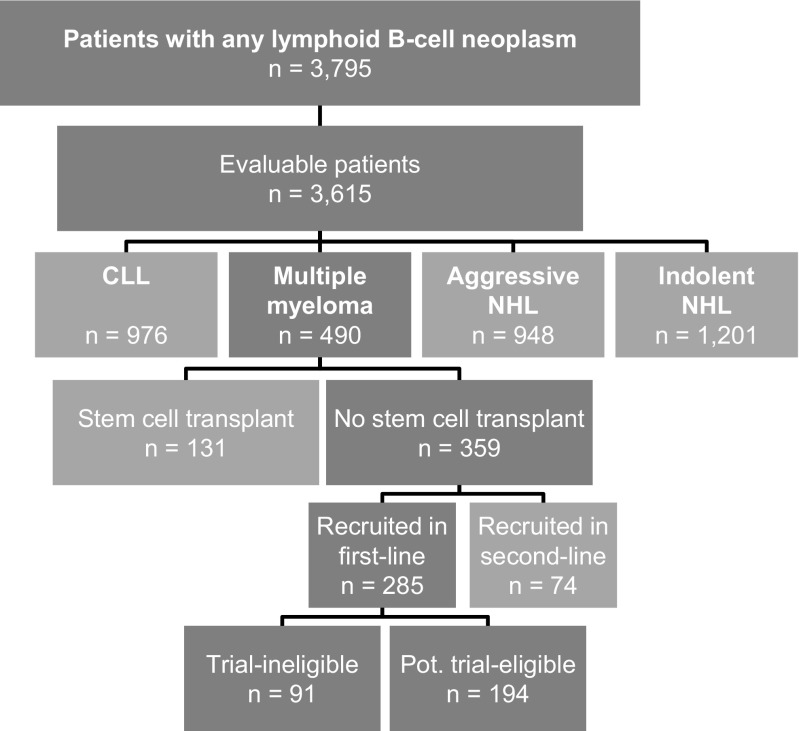
Cohort definition. Number of patients enrolled in the TLN, split up according to the four different types of lymphoid B cell neoplasms. Patients with multiple myeloma were recruited between April 2009 and November 2011 and followed until August 2016. This analysis focuses on the patients with multiple myeloma, who did not receive a stem cell transplant, and whose first-line treatment was prospectively documented (enrolment at start of first-line treatment, *n* = 285). Abbreviations: CLL, chronic lymphocytic leukaemia; NHL, non-Hodgkin lymphoma; pot, potentially

At data cut in August 2016, 3795 patients with lymphoid B cell neoplasms had been recruited into the TLN; 180 patients had incomplete basic medical data or withdrew consent. Of the 3615 evaluable patients, 490 were diagnosed with MM and 359 of them had not received a stem cell transplant. The present analysis focuses on 285 non-transplant patients with MM recruited at the start of first-line treatment (Fig. 1).

### Definition of trial-ineligibility and -eligibility

The potential eligibility for clinical trials was defined according to common exclusion criteria in phase III clinical trials and explicitly captured by the TLN registry. Patients were classified as trial-ineligible when at least one of the following exclusion criteria had been fulfilled: heart failure, renal failure, other renal diseases, liver diseases, polyneuropathy, and HIV positivity. When none of these criteria were fulfilled, patients were classified as potentially trial-eligible. Detailed eligibility classification rules have been described before [16]. The exclusion criteria in phase III clinical trials are often limited to a certain grade/severity; as grade or severity of parameters (especially of polyneuropathy and renal failure) were not documented in the registry, all patients with documented polyneuropathy or renal failure were classified as trial-ineligible.

### Statistical analysis

All analyses were performed using Statistica Version 13.1 and SAS for Windows version 9.4 (Copyright© 2002–2012 SAS Institute Inc., Cary, NC, USA). Time to events was analysed using the Kaplan-Meier method. PFS was defined as the interval between start of first-line therapy and the date of first progression or death, whatever came first. Data of patients without such an event before start of second-line therapy were censored at either the start of second-line therapy or at last contact, whatever came first. OS was defined as the interval between start of first-line therapy and the date of death from any cause. Data of patients alive or lost to follow-up were censored at last contact. DSS was calculated from start of first-line therapy until date of death due to MM. Data of patients alive, lost to follow-up, or with other causes of death were censored at last contact or at date of death, whatever came last.

## Results

### Patient, tumour, and first-line treatment characteristics

Table 1 presents the patient and tumour characteristics of the 285 patients included into this analysis. Thirty-two percent (*n* = 91) of all patients were classified as trial-ineligible as explained by the criteria listed in the methods section. Median age at start of therapy was 74 years; trial-ineligible patients were older (76 years, range 56–92) than potentially trial-eligible patients (73 years, range 42–90 years). Prominent differences between the patient subgroups were the frequency and burden of comorbidities: at least one comorbid condition was recorded for 100% of the trial-ineligible (74% CCI ≥ 1) and 70% of the trial-eligible patients (19% CCI ≥ 1). While the staging according to Durie Salmon was similar for both subgroups, more trial-ineligible patients were diagnosed in prognostic stage III according to the ISS criteria (32 vs 10%). 59% patients (65%) were classified as trial-ineligible due to one exclusion criterion, 23 patients (25%) met two exclusion criteria, eight patients (9%) met three, and one patient (1%) met four exclusion criteria.

**Table 1:** Patient and tumour characteristics

Characteristic	Trial-ineligible (*n* = 91)		Pot. trial-eligible (*n* = 194)		All patients (*n* = 285)	
	Years	Min–max	Years	Min–max	Years	Min–max
Median age at start of therapy	76.4	56.2–92.0	72.5	41.6–90.1	73.7	41.6–92.0
	Mean	SD	Mean	SD	Mean	SD
BMI at enrolment, kg/m^2^ (*n* = 278)	25.7	3.9	26.3	4.4	26.1	4.3
Sex	*n*	%	*n*	%	*n*	%
Female	36	39.6%	107	55.2%	143	50.2%
Male	55	60.4%	87	44.8%	142	49.8%
Patients with any comorbidity^a, b, c^	91	100%	136	70.1%	227	79.6%
Diabetes mellitus without diabetic compl.	20	22.0%	22	11.3%	42	14.7%
Diabetes mellitus with diabetic compl.	5	5.5%	4	2.1%	9	3.2%
Heart failure	29	31.9%	0	0%	29	10.2%
Liver disease (mild)	5	5.5%	0	0%	5	1.8%
Liver disease (moderate or severe)	3	3.3%	0	0%	3	1.1%
Renal disease (moderate or severe)	30	33.0%	0	0%	30	10.5%
Renal failure	55	60.4%	0	0%	55	19.3%
Polyneuropathy	10	11.0%	0	0%	10	3.5%
AIDS	1	1.1%	0	0%	1	0.4%
Charlson Comorbidity Index^a, c^						
CCI = 0	24	26.4%	157	80.9%	181	63.5%
CCI ≥ 1	67	73.6%	37	19.1%	104	36.5%
Durie Salmon stage^a^						
I	9	9.9%	14	7.2%	23	8.1%
II	22	24.2%	45	23.2%	67	23.5%
III	51	56.0%	119	61.3%	170	59.6%
Unknown/missing	9	9.9%	16	8.2%	25	8.8%
ISS stage^a^						
I	6	6.6%	45	23.2%	51	17.9%
II	15	16.5%	63	32.5%	78	27.4%
III	29	31.9%	20	10.3%	49	17.2%
Unknown^d^	41	45.1%	66	34.0%	107	37.5%
B symptoms^a^	11	12.1%	25	12.9%	36	12.6%

^a^At the start of therapy

^b^At least one comorbidity according to Charlson [19] or additional concomitant diseases

^c^Charlson Comorbidity Index (CCI) according to Quan et al. [20]

^d^For some patients, the exact stage according to ISS could not be determined because of unknown parameters (β2-microglobulin or albumin)

Abbreviations: BMI, body mass index; compl., complications; ISS, international staging system; max, maximum; min, minimum; SD, standard deviation

Table 2 presents the first-line treatment characteristics for the patient subgroups and all patients. The majority of the patients received a proteasome inhibitor (PI): 81% of the trial-ineligible and 73% of the trial-eligible patients, while approximately 10% of the patients in both groups received an immunomodulatory drug (IMiD). The most frequently applied first-line regimen was a combination of bortezomib and melphalan, with or without additional prednisone (given to 43% of the trial-ineligible and 41% of the potentially trial-eligible patients). Trial-ineligible patients more often received bortezomib monotherapy (13% vs. 6%).

**Table 2:** First-line treatment characteristics

Characteristic	Trial-ineligible (*n* = 91)		Pot. trial-eligible (*n* = 194)		All patients (*n* = 285)	
	*n*	%	*n*	%	*n*	%
First-line treatment strategy						
Proteasome inhibitor (PI)	74	81.3%	141	72.7%	215	75.4%
Immunomodulatory drug (IMiD)	9	9.9%	18	9.3%	27	9.5%
Combination therapy (PI + IMiD)	0	0.0%	2	1.0%	2	0.7%
Other	8	8.8%	33	17.0%	41	14.4%
Main first-line regimen^a^						
Bor + Mel ± Pre (VMP)	39	42.9%	80	41.2%	119	41.8%
Bor + Dex (V + D)	14	15.4%	32	16.5%	46	16.1%
Bor (V(mono))	12	13.2%	12	6.2%	24	8.4%
Mel + Pre + Tha (MPT)	9	9.9%	15	7.7%	24	8.4%
Mel + Pre (MP)	4	4.4%	10	5.2%	14	4.9%
Bor + Dex + Dox (VDD)	2	2.2%	10	5.2%	12	4.2%
Ben ± Pre B(P)	2	2.2%	10	5.2%	12	4.2%

^a^All treatments with at least 10 documented cases overall were listed. Patients received first-line treatment between 2009 and 2011

Abbreviations: Ben, bendamustine; Bor, bortezomib; Dex, dexamethason; Dox, doxorubicin; IMiD, immunomodulatory drug; Mel, melphalan; PI, proteasome inhibitor; pot, potentially; Pre, prednisone; Tha, thalidomide

### Sequential lines of treatment

**Fig. 2:**
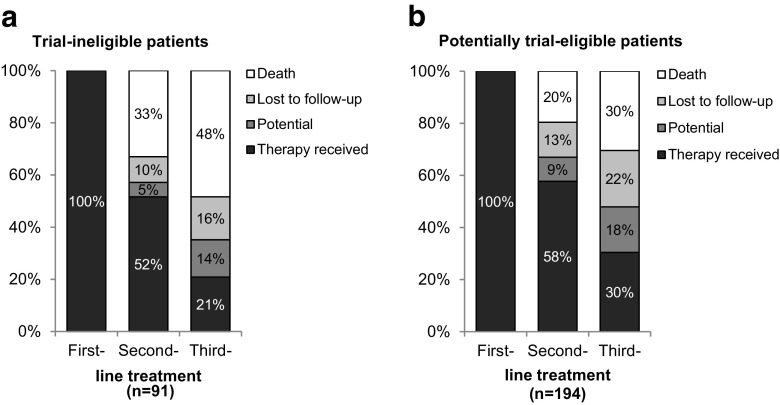
Sequential lines of treatment. Sequential lines of treatments in **a** trial-ineligible and **b** potentially trial-eligible patients. The category “potential” includes patients with ongoing or paused line of treatment and patients who had completed the 5-year follow-up period

52% (21%) of the trial-ineligible and 58% (30%) of the potentially trial-eligible patients received a second-line (third-line) treatment (Fig. 2). 48% of the trial-ineligible patients died before receiving a third-line treatment (Fig. 2a), compared to 30% of the potentially trial-eligible patients (Fig. 2b). Patients marked as “potential” for another line of treatment had either not yet completed the previous line of treatment or had finished the previous line but not yet started a new one or had completed the 5-year follow-up period.

### Second-line treatment

**Fig. 3:**
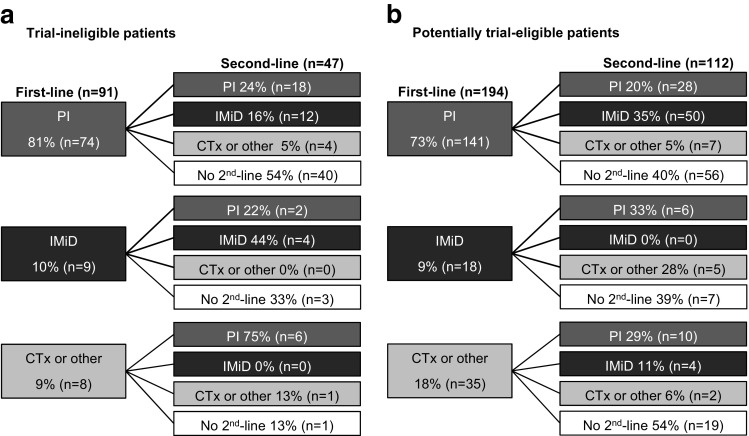
Second-line treatment of trial-ineligible and trial-eligible patients. Sequencing of treatments in **a** trial-ineligible and **b** potentially trial-eligible patients. Percentages in second-line treatment refer to all patients who had received the respective first-line treatment. The treatment group “CTx or other” includes chemotherapy as well as all other treatments, including combination therapies. Abbreviations: CTx, chemotherapy; IMiD, immunomodulatory drug; PI, proteasome inhibitor

Looking at the sequencing of treatments, a variety of sequential treatment strategies was employed, mostly PI- and IMiD-based, both in trial-ineligible patients (Fig. 3a) and potentially trial-eligible patients (Fig. 3b).

### Patient outcomes

**Fig. 4:**
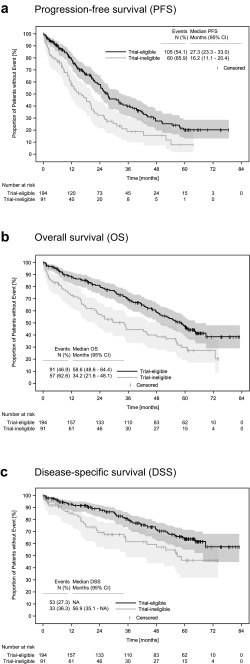
Survival of potentially trial-eligible patients compared to trial-ineligible patients. Survival analysis showing the **a** PFS, **b** OS, and **c** DSS for the potentially trial-eligible and -ineligible patients. Abbreviations: CI, confidence interval; DSS, disease-specific survival; NA, not available (not reached); OS, overall survival; PFS, progression-free survival

Regardless of the type of treatment, median first-line PFS of the potentially trial-eligible patients was markedly longer than that of the trial-ineligible patients: 27.3 months, 95% CI (confidence interval) 23.3–33.0 vs. 16.2 months, 95% CI 11.1–20.4 (Fig. 4a). The 3-year PFS rate was 39.9% for potentially trial-eligible patients (95% CI 31.6–48.1%) and 18.8% for trial-ineligible patients (95% CI 9.8–29.9%). At the time of this analysis, 148 patients (52%) had died, corresponding to 47% of the potentially trial-eligible and 63% of the trial-ineligible patients. Median OS for the whole cohort was 52.0 months (95% CI 43.5–58.7, data on file). Median OS was considerably longer for potentially trial-eligible patients: 58.6 months (95% CI 48.6–64.4) than for trial-ineligible patients: 34.2 months (95% CI 21.6–48.1, Fig. 4b). The 3-year OS rate was 69.4% for potentially trial-eligible patients (95% CI 61.9–75.7%) and 44.4% for trial-ineligible patients (95% CI 33.3–55.0%).

MM was documented as cause of death for 33 (35%) trial-ineligible and 53 (27%) potentially trial-eligible patients. Comorbidities were documented as cause of death for seven (8%) of the trial-ineligible and eight (4%) of the potentially trial-eligible patients. The cause of death was unknown to the oncologist for 11 (12%) of the trial-ineligible and 20 (10%) of the trial-eligible patients. DSS was not reached for the potentially trial-eligible patients (Fig. 4c). The 2-year DSS rate was 88.4% (95% CI 82.6–92.4) for the potentially trial-eligible and 69.3% (95% CI 57.3–78.5) for the trial-ineligible patients.

## Discussion

Patients in clinical trials are highly selected and might not be representative of all patients with MM; thus, outcome results may not be conferrable to the routine clinical practice. Here, we present data from the prospective, multicentre, clinical registry TLN, showing that at least 30% of the non-transplant patients with MM would be deemed ineligible for clinical trials. These patients showed inferior survival, although the treatment algorithm was similar. This highlights the need for cohort studies with patients in routine care and for an individualised therapy coupled with recommendations for such an approach. For the potentially trial-eligible patients, outcome was similar to the survival data from clinical trials.

Strengths of this study are the prospective, longitudinal design and the participation of haematologists/oncologists across Germany. This study is limited by the sole enrolment of patients receiving systemic therapy, excluding patients under “watch and wait,” patients refusing such treatment as well as frail patients not able to receive systemic therapy or patients receiving local therapy (surgery, radiotherapy) only. Due to the observational design of this registry study, there is no randomisation of treatment or study sites and thus causal relations cannot be drawn. Trial ineligibility was defined using frequently applied exclusion criteria from phase III clinical trials. The proportion of trial-ineligible patients may be underestimated because not all data for exclusion criteria from clinical trials had been collected in the TLN registry. On the other hand, all patients with polyneuropathy or renal insufficiency were excluded, regardless of the grade/severity, possibly overestimating the proportion of trial-ineligible patients. There are no specifications as to the timing, frequency, or criteria of tumour assessment; thus, clinical PFS data should be considered as the best clinical approximation and might not be identical to the PFS determined in clinical trials.

In the field of MM, the approval of novel agents has significantly advanced and improved the range of therapeutic options and the clinical outcome. Median survival times of more than 4 years have been achieved, leading to a paradigm shift in treatment, also for non-transplant, elderly patients (reviewed in [14]). However, these results are mainly derived from clinical trials enrolling a highly selected patient population. Are the results conferrable to the patients in routine care? For non-transplant patients, the VISTA trial reported a median OS of 56.4 months for patients receiving VMP [21], and the FIRST trial reported a median OS of 48.5 months for patients treated in the MPT arm [22]. In our cohort, median OS was 52.0 months with 42% of the patients receiving VMP. While the survival data for the potentially trial-eligible patients in our analysis (median OS 58.6 months) were similar to the survival times reported in these phase III clinical trials, trial-ineligible patients had inferior outcomes (median OS 34.2 months) despite similar first-line treatment and a similar proportion of patients receiving second-line treatment (52 vs. 58%). Related findings of inferior outcomes for patients not fulfilling the criteria for trial eligibility have been reported for other malignancies like metastatic colorectal cancer [23] or metastatic renal cell carcinoma [15, 16]. The difference in survival times between potentially trial-eligible and -ineligible patients appeared smaller when DSS was analysed, although these data have to be considered preliminary due to the small number of events. This might implicate that not only the malignancy but also other factors like the comorbidities influence the survival of trial-ineligible patients. Aside from specific disease characteristics, there are multiple known prognostic factors for not only survival in patients with MM: foremost age but also performance status and prevalence of comorbidities like renal or lung disease [24–28]. The frailty score of the International Myeloma Working Group predicting survival and toxicities is based on age, comorbidities, as well as cognitive and physical conditions [29], and the revised Myeloma Comorbidity Index incorporates renal, lung, and Karnofsky performance status impairment, frailty, and age as significant risks for OS [28].

Besides HIV positivity, all factors used in our study to determine trial eligibility are known prognostic factors for survival. Hence, the patients enrolled in clinical trials are those with a favourable prognosis, resulting in approval of treatment algorithms fitted to their needs. For example, no changes in dose or schedule of MPT or VMP are approved according to age or performance status, although these regimens are accompanied by a high rate of grade 3–4 adverse events [30, 31]. A study on 502 non-transplant patients in US community practice showed no advantage of triplet combinations over the doublet regimen VD [32], highlighting the need for adapted treatment algorithms for elderly and trial-ineligible patients.

The immunomodulatory drug lenalidomide combined with low-dose dexamethasone has shown promising efficacy and safety results [8] and has been approved in EU for first-line treatment of non-transplant patients in February 2015. This regimen is also a backbone for combinations with carfilzomib, ixazomib, or elotuzumab [14]. This may well pave the way for an alkylator-free new standard of care for elderly patients. It will be interesting to see the incorporation of these novel agents in routine practice and follow their effectiveness. Our successor cohort study, recruiting patients with MM in routine care in Germany was started in 2017 (ClinicalTrials.gov NCT03308474).

## Conclusion

Our study addresses the recent demand for validation of results from clinical trials in the routine care setting [18]. We show that at least 30% of the patients in daily routine care would be deemed ineligible for clinical trials. Their first-line PFS and OS were inferior to those of potentially trial-eligible patients, while the survival times from these potentially trial-eligible patients were comparable to the survival times reported by clinical trials. The patient population selected for clinical trials specifically excludes patients with an inferior prognosis and thus data from prospective, multicentre, clinical registries like ours are of paramount importance to complement these data. In addition, clinical trials specifically addressing this commonly excluded comorbid group of patients are needed to develop optimised treatment strategies.
